# A nomogram based on serum bilirubin and albumin levels predicts survival in gastric cancer patients

**DOI:** 10.18632/oncotarget.17181

**Published:** 2017-04-18

**Authors:** Huiling Sun, Bangshun He, Zhenlin Nie, Yuqin Pan, Kang Lin, Hongxin Peng, Tao Xu, Xiaoxiang Chen, Xiuxiu Hu, Zijuan Wu, Di Wu, Shukui Wang

**Affiliations:** ^1^ General Clinical Research Center, Nanjing First Hospital, Nanjing Medical University, Nanjing, 210006, China; ^2^ Laboratory Medicine, Nanjing First Hospital, Nanjing Medical University, Nanjing, 210006, China; ^3^ School of Medicine, Southeast University, Nanjing, 210009, China; ^4^ First Affiliated Hospital, Nanjing Medical University, Nanjing, 210029, China; ^5^ School of Medicine, Yangzhou Occupational University, Yangzhou, 225009, China

**Keywords:** gastric cancer, bilirubin, albumin, prognosis, nomogram

## Abstract

Decreases in serum bilirubin and albumin levels are associated with poorer prognoses in some types of cancer. Here, we examined the predictive value of serum bilirubin and albumin levels in 778 gastric cancer patients from a single hospital in China who were divided among prospective training and retrospective validation cohorts. X-tile software was used to identify optimal cutoff values for separating training cohort patients into higher and lower overall survival (OS) groups, based on total bilirubin (TBIL) and albumin levels. In univariate analysis, tumor grade and TNM stage were associated with OS. After adjusting for tumor grade and TNM stage, TBIL and albumin levels were still clearly associated with OS. These results were confirmed in the 299 patients in the validation cohort. A nomogram based on TBIL and albumin levels was more accurate than the TNM staging system for predicting prognosis in both cohorts. These results suggest that serum TBIL and albumin levels are independent predictors of OS in gastric cancer patients, and that an index that combines TBIL and albumin levels with the TNM staging system might have more predictive value than any of these measures alone.

## INTRODUCTION

Gastric cancer is the second most common malignant disease and cause of cancer-related death worldwide, especially in developing countries [1]. Predicting clinical outcomes for gastric cancer patients can be challenging due to genetic and geographically-dependent differences in incidence, stage at treatment, and patient prognosis [2]. However, accurate prediction of prognosis is crucial for selecting therapeutic strategies and for effective communication between doctors and gastric cancer patients.

Generally, prognostic evaluations in gastric cancer are based mainly on the tumor-node-metastasis (TNM) staging system. However, due to constraints associated with maintaining its simplicity and uniformity, the TNM staging system does not take into account many essential variables, including patient characteristics, laboratory test results, and treatments administered, that can influence the survival of gastric cancer patients [3]. The TNM staging system is therefore limited in its ability to assess survival in these patients, and survival predictions can vary widely for each gastric cancer stage [4]. Nomograms have been increasingly used to more accurately predict individualized patient outcomes in a variety of cancers. However, few nomograms have been developed that predict clinical outcomes for gastric cancer patients.

Serum bilirubin, which is an end product of heme metabolism, was not thought to serve any physiological function. Nevertheless, recent studies have demonstrated that serum bilirubin has potent antioxidant, anti-inflammatory, and anticancer effects in colorectal cancer [5, 6]. The protective effects of serum bilirubin have been reported in lung cancer [7, 8], colorectal cancer [9], and breast cancer [10]. Additionally, decreased levels of albumin, a factor commonly used as an indicator of nutritional status, are associated with worse outcomes in gastric cancer patients [11].

Although they might improve prognostic predictions in gastric cancer, no measurements or indices that combine serum bilirubin and albumin levels have been developed. In this study, we examined whether serum bilirubin and albumin levels were predictive of survival outcomes in gastric cancer patients. We then evaluated the predictive value of a nomogram based on serum bilirubin and albumin levels in these patients.

## RESULTS

### Baseline characteristics

Baseline characteristics for the two patient cohorts are summarized in Table 1. A total of 352 men and 127 women were prospectively enrolled in the training cohort. Of these patients, 180 (37.6%) had distant metastasis and 275 (57.4%) received chemotherapy. During the follow-up period, 298 (62.2%) patients died (median follow-up, 1156 days). The median values for TBIL, DBIL, IDBIL, and albumin were 10.1 μmol/L, 3.6 μmol/L, 6.3 μmol/L, and 37.1 g/L, respectively. An additional 299 patients were retrospectively enrolled in the validation cohort. Pathologic TNM staging distributions varied widely; 31 (10.4%) individuals had early gastric cancer, and 72 (24.1%) had node negative disease. Two hundred six had died (median follow-up, 989 days) during the follow-up period.

**Table 1:** Baseline characteristics for training and validation cohort patients

Clinical characteristics	Training cohort	Validation cohort	*P*
Age	65 (57-74)	66 (57-74)	0.756
Gender (male)	352 (73.5)	220 (73.6)	0.977
Smoking	110 (23.0)	56 (18.7)	0.161
Drinking	52 (10.9)	59 (19.7)	0.001
*Helicobator pylori*	261 (54.5)	172 (57.5)	0.407
Tumor differentiation (well/moderate/poor)	89/194/196	40/140/119	0.092
pT stage (1/2/3/4)	77/49/199/154	31/25/142/101	0.085
pN stage (0/1/2/3)	125/141/97/116	72/110/56/61	0.189
Metastasis	180 (37.6)	127 (42.5)	0.174
Chemotherapy	275 (57.4)	163 (54.5)	0.428
Curative/palliative	285/194	161/138	0.121
TBIL (μmol/L)	10.1 (7.1-14.6)	9.9 (6.4-13.9)	0.411
DBIL (μmol/L)	3.6 (2.5-5.2)	3.5 (2.0-5.4)	0.123
IBIL (μmol/L)	6.3 (4.3-9.4)	6.2 (3.5-9.3)	0.078
Albumin (g/L)	37.1 (34.1-40.5)	37.2 (33.7-40.0)	0.544

TBIL, Total bilirubin; DBIL, direct bilirubin; IBIL, indirect bilirubin.

Values are medians (interquartile range) or frequencies and percentages.

Patients received treatment for either curative or palliative purposes according to the Japanese Classification of Gastric Cancer Guidelines.

Data were analyzed using χ^2^ test or Mann-Whitney *U* test.

### Associations between TBIL, DBIL, IBIL, and albumin levels and clinical characteristics

**Figure 1:**
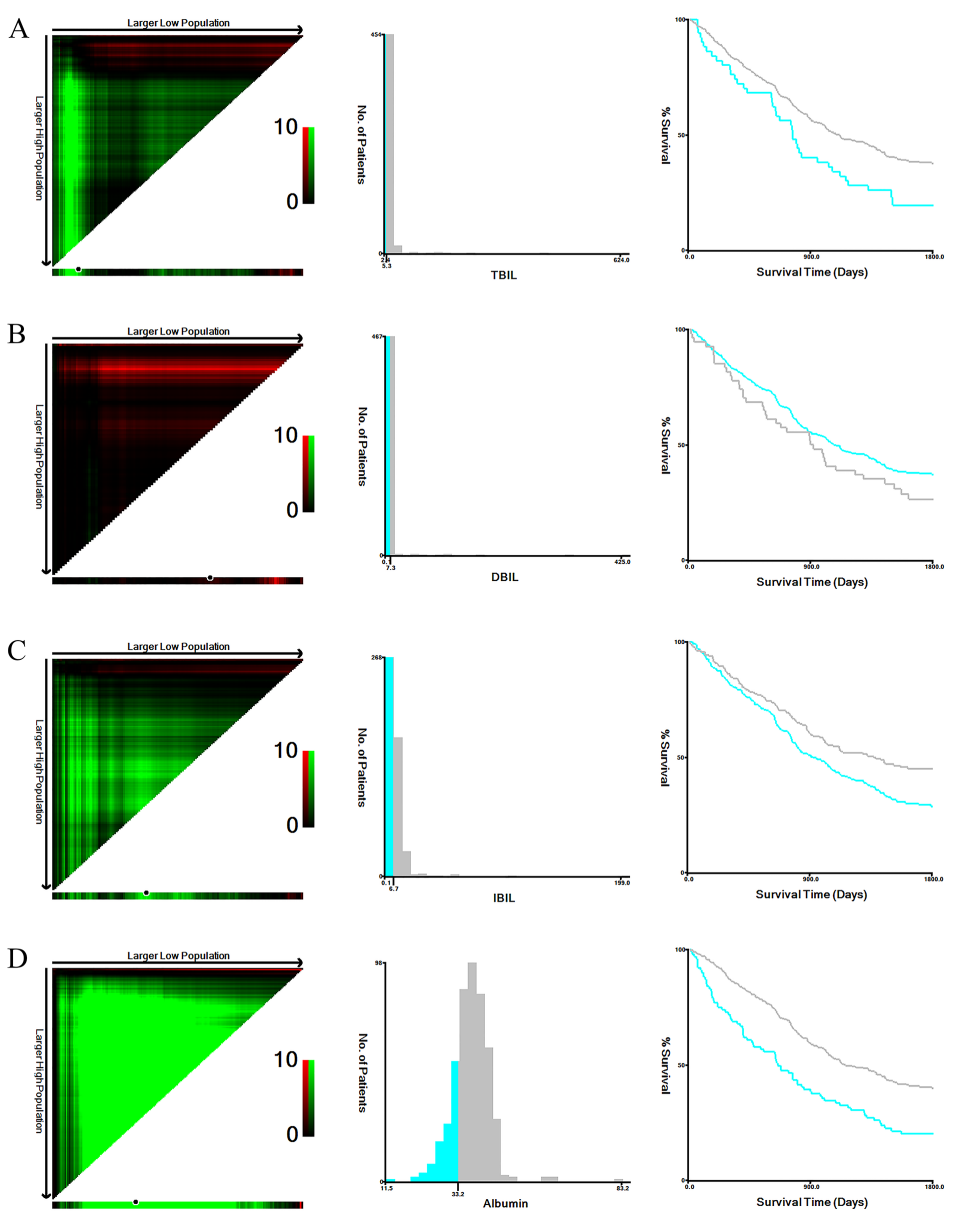
X-tile analyses of TBIL (A), DBIL (B), IBIL (C), and albumin (D) levels in training cohort gastric cancer patients X-tile plots for training cohort patients are shown in the left panels; black circles highlight the optimal cutoff values, which are also shown in histograms (middle panels). Kaplan-Meier plots are presented in right panels.

We first compared clinical characteristics of training and validation cohort patients. Patients in the two cohorts were similar with regard to all clinical characteristics except drinking (*P*<0.001) (Table 1), indicating that the cohorts were comparable. We then explored associations between TBIL, DBIL, IBIL, and albumin levels and clinical characteristics. X-tile software was used to determine the optimal cutoff values of 5.3 μmol/L for TBIL, 7.3μmol/L for DBIL, 6.7 μmol/L for IBIL, and 33.2 g/L for albumin based on OS in training cohort patients (Figure 1). Training cohort patients were then divided into high and low level groups based on these cutoffs. Lymph node metastasis (N2 and N3 stage) was more common in patients in the low TBIL group than in those in the high TBIL group (Table 2). In addition, markedly more patients in the high DBIL group had T4 and N3 stage disease compared to the low DBIL group (Table 3). T1 stage and N0 stage disease were more common in patients in the high IBIL group than in those in the low IBIL group (Table 4). Finally, patients in the high albumin group were younger and more likely to have T1-T2, N0, or M0 stage disease than those in low albumin group (Table 5). Similar results were obtained when validation cohort patients were divided into high and low level groups for each measure based on the same optimal cutoff values used for the training cohort (Table 2-5).

**Table 2:** Associations between TBIL level and clinical characteristics in training and validation cohort patients

Variable	TBIL group					
	Training cohort			Validation cohort		
	Low TBIL	High TBIL	*P*	Low TBIL	High TBIL	*P*
Age	63 (57-75)	65 (58-74)	0.779	66.5 (57-78)	65 (57-74)	0.173
Gender						
Male	36 (70.6)	316 (73.8)	0.620	41 (73.2)	179 (73.7)	0.945
Female	15 (29.4)	112 (26.2)		15 (26.8)	64 (26.3)	
Smoking						
Never	39 (76.5)	330 (77.1)	0.919	44 (78.6)	199 (81.9)	0.566
Yes	12 (23.5)	98 (22.9)		12 (21.4)	44 (18.1)	
Drinking						
Never	43 (84.3)	384 (89.7)	0.241	45 (80.4)	195 (80.2)	0.985
Yes	8 (15.7)	44 (10.3)		11 (19.6)	48 (19.8)	
*Helicobator pylori*						
Negative	21 (41.2)	197 (46.0)	0.511	25 (44.6)	102 (42.0)	0.716
Positive	30 (58.8)	231 (54.0)		31 (55.4)	141 (58.0)	
Differentiation						
Well	8 (15.7)	81 (18.9)	0.591	6 (10.7)	34 (14.0)	0.127
Moderate	24 (47.1)	170 (39.7)		21 (37.5)	119 (49.0)	
Poor	19 (37.3)	177 (41.4)		29 (51.8)	90 (37.0)	
pT stage						
T1	5 (9.8)	72 (16.8)	0.400	1 (1.8)	30 (12.3)	0.001
T2	4 (7.8)	45 (10.5)		2 (3.6)	23 (9.5)	
T3	26 (51.0)	173 (40.4)		23 (41.1)	119 (49.0)	
T4	16 (31.4)	138 (32.2)		30 (53.6)	71 (29.2)	
pN stage						
N0	10 (19.6)	115 (26.9)	0.017	14 (25.0)	58 (23.9)	0.043
N1	12 (23.5)	129 (30.1)		12 (21.4)	98 (40.3)	
N2	19 (37.3)	78 (18.2)		14 (25.0)	42 (17.3)	
N3	10 (19.6)	106 (24.8)		16 (28.6)	45 (18.5)	
Metastasis						
Absent	30 (58.8)	269 (62.9)	0.575	27 (48.2)	145 (59.7)	0.117
Present	21 (41.2)	159 (37.1)		29 (51.8)	98 (40.3)	
Chemotherapy						
No	28 (54.9)	176 (41.1)	0.060	30 (53.6)	106 (43.6)	0.178
Yes	23 (45.1)	252 (58.9)		26 (46.4)	137 (56.4)	
Treatments						
Curative	28	257	0.479	25	136	0.125
Palliative	23	171		31	107	

TBIL, total bilirubin.

Values are medians (interquartile range) or frequencies and percentages.

Data were analyzed using χ^2^ test or Mann-Whitney *U* test.

**Table 3:** Associations between DBIL levels and clinical characteristics in training and validation cohort patients

Variable	DBIL group					
	Training cohort			Validation cohort		
	Low DBIL	High DBIL	*P*	Low DBIL	High DBIL	*P*
Age	65 (58-74)	63 (54-74)	0.598	66 (57-74)	62 (52-75)	0.429
Gender						
Male	308 (72.0)	44 (86.3)	0.029	197 (73.0)	23 (79.3)	0.663
Female	120 (28.0)	7 (13.7)		73 (27.0)	7 (20.7)	
Smoking						
Never	331 (77.3)	38 (74.5)	0.650	219 (81.1)	24 (82.8)	0.829
Yes	97 (22.7)	13 (25.5)		51 (18.9)	5 (17.2)	
Drinking						
Never	381 (89.0)	46 (90.2)	0.798	218 (80.7)	22 (75.9)	0.530
Yes	47 (11.0)	5 (9.8)		52 (19.3)	7 (24.1)	
*Helicobator pylori*						
Negative	193 (45.1)	25 (49.0)	0.595	116 (43.0)	11 (37.9)	0.602
Positive	235 (54.9)	26 (51.0)		154 (57.0)	18 (62.1)	
Differentiation						
Well	82 (19.2)	7 (13.7)	0.529	32 (11.9)	8 (27.6)	0.059
Moderate	174 (40.7)	20 (39.2)		128 (47.4)	12 (41.4)	
Poor	172 (40.2)	24 (47.1)		110 (40.7)	9 (31.0)	
pT stage						
T1	65 (15.2)	12 (23.5)	0.009	30 (11.1)	1 (3.4)	0.014
T2	46 (10.7)	3 (5.9)		20 (7.4)	5 (17.2)	
T3	187 (43.7)	12 (23.5)		134 (49.6)	8 (27.6)	
T4	130 (30.4)	24 (47.1)		86 (31.9)	15 (51.7)	
pN stage						
N0	112 (26.2)	13 (25.5)	0.005	69 (25.6)	3 (10.3)	0.003
N1	135 (31.5)	6 (11.8)		104 (38.6)	6 (20.7)	
N2	86 (20.1)	11 (21.6)		49 (18.1)	7 (24.1)	
N3	95 (22.2)	21 (41.2)		48 (17.7)	13 (44.8)	
Metastasis						
Absent	273 (63.8)	26 (51.0)	0.074	157 (58.1)	15 (51.7)	0.506
Present	155 (36.2)	25 (49.0)		113 (41.9)	14 (48.3)	
Chemotherapy						
No	183 (42.8)	21 (41.2)	0.829	118 (43.7)	18 (62.1)	0.059
Yes	245 (57.2)	30 (58.8)		152 (56.3)	11 (37.9)	
Treatments						
Curative	259	26	0.190	147	14	0.527
Palliative	169	25		123	15	

DBIL, direct bilirubin.

Values are medians (interquartile range) or frequencies and percentages.

Data were analyzed using χ^2^ test or Mann-Whitney *U* test.

**Table 4:** Associations between IBIL levels and clinical characteristics in training and validation cohort patients

Variable	IBIL group					
	Training cohort			Validation cohort		
	Low IBIL	High IBIL	*P*	Low IBIL	High IBIL	*P*
Age	66 (59-76)	64 (55-71)	0.078	66 (57-75)	64 (56-73)	0.349
Gender						
Male	188 (72.3)	164 (74.9)	0.524	131 (77.1)	89 (69.0)	0.117
Female	72 (27.7)	55 (25.1)		39 (22.9)	40 (31.0)	
Smoking						
Never	203 (78.1)	166 (75.8)	0.555	139 (81.8)	104 (80.6)	0.802
Yes	57 (21.9)	53 (24.2)		31 (18.2)	25 (19.4)	
Drinking						
Never	233 (89.6)	194 (88.6)	0.718	133 (78.2)	107 (82.9)	0.311
Yes	27 (10.4)	25 (11.4)		37 (21.8)	22 (17.1)	
*Helicobator pylori*						
Negative	110 (42.3)	108 (49.3)	0.125	72 (42.4)	55 (42.6)	0.961
Positive	150 (57.7)	111 (50.7)		98 (57.6)	74 (57.4)	
Differentiation						
Well	44 (16.9)	45 (20.5)	0.593	28 (16.5)	12 (9.3)	0.069
Moderate	108 (41.5)	86 (39.3)		71 (41.8)	69 (53.5)	
Poor	108 (41.5)	88 (40.2)		71 (41.8)	48 (37.2)	
pT stage						
T1	28 (10.8)	49 (22.4)	0.006	10 (5.9)	21 (16.3)	0.013
T2	26 (10.0)	23 (10.5)		15 (8.8)	10 (7.8)	
T3	115 (44.2)	84 (38.4)		79 (46.5)	63 (48.8)	
T4	91 (35.0)	63 (28.8)		66 (38.8)	35 (27.1)	
pN stage						
N0	54 (20.8)	71 (32.4)	0.011	36 (21.2)	36 (27.9)	0.003
N1	83 (31.9)	58 (26.5)		69 (40.6)	41 (31.8)	
N2	62 (23.8)	35 (16.0)		40 (23.5)	16 (12.4)	
N3	61 (23.5)	55 (25.1)		25 (14.7)	36 (27.9)	
Metastasis						
Absent	152 (58.5)	147 (67.1)	0.051	96 (56.5)	76 (58.9)	0.672
Present	108 (41.5)	72 (32.9)		74 (43.5)	53 (41.1)	
Chemotherapy						
No	115 (44.2)	89 (40.6)	0.428	80 (47.1)	56 (43.4)	0.530
Yes	145 (55.8)	130 (59.4)		90 (52.9)	73 (56.6)	
Treatments						
Curative	150	135	0.380	88	73	0.407
Palliative	110	84		82	56	

IBIL, indirect bilirubin.

Values are medians (interquartile range) or frequencies and percentages.

Data were analyzed using χ^2^ test or Mann-Whitney *U* test.

**Table 5:** Association between albumin levels and clinical characteristics in training and validation cohort patients

Variable	Albumin group					
	Training cohort			Validation cohort		
	Low Albumin	High Albumin	*P*	Low Albumin	High Albumin	*P*
Age	72 (63.5-78)	64 (56-71)	<0.001	74 (64-78)	64 (56-72)	<0.001
Gender						
Male	71 (70.3)	281 (74.3)	0.414	50 (76.9)	170 (72.6)	0.489
Female	30 (29.7)	97 (25.7)		15 (23.1)	64 (27.4)	
Smoking						
Never	79 (78.2)	290 (76.7)	0.750	54 (83.1)	189 (80.8)	0.673
Yes	22 (21.8)	88 (23.3)		11 (16.9)	45 (19.2)	
Drinking						
Never	91 (90.1)	336 (88.9)	0.728	56 (86.2)	184 (78.6)	0.178
Yes	10 (9.9)	42 (11.1)		9 (13.8)	50 (21.4)	
*Helicobator pylori*						
Negative	48 (47.5)	213 (56.3)	0.114	23 (35.4)	104 (44.4)	0.191
Positive	53 (52.5)	165 (43.7)		42 (64.6)	130 (55.6)	
Differentiation						
Well	15 (14.9)	74 (19.6)	0.275	9 (13.8)	31 (13.2)	0.922
Moderate	38 (37.6)	156 (41.3)		29 (44.6)	111 (47.4)	
Poor	48 (47.5)	148 (39.2)		27 (41.5)	92 (39.3)	
pT stage						
T1	8 (7.9)	69 (18.3)	<0.001	1 (1.5)	30 (12.8)	<0.001
T2	7 (6.9)	42 (11.1)		2 (3.1)	23 (9.8)	
T3	36 (35.6)	163 (43.1)		28 (43.1)	114 (48.7)	
T4	50 (49.5)	104 (27.5)		34 (52.3)	67 (28.6)	
pN stage						
N0	15 (14.9)	110 (29.1)	<0.001	9 (13.8)	63 (26.9)	0.042
N1	23 (22.8)	118 (31.2)		27 (41.5)	83 (35.5)	
N2	30 (29.7)	67 (17.7)		18 (27.7)	38 (16.2)	
N3	33 (32.7)	83 (22.0)		11 (16.9)	50 (21.4)	
Metastasis						
Absent	42 (41.6)	257 (68.0)	<0.001	23 (35.4)	149 (63.7)	<0.001
Present	59 (58.4)	121 (32.0)		42 (64.6)	85 (36.3)	
Chemotherapy						
No	50 (49.5)	154 (40.7)	0.114	36 (55.4)	100 (42.7)	0.070
Yes	51 (50.0)	224 (59.3)		29 (44.6)	134 (57.3)	
Treatments						
Curative	53	232	0.106	30	131	0.160
Palliative	48	146		35	103	

Values are medians (interquartile range) or frequencies and percentages.

Data were analyzed using χ^2^ test or Mann-Whitney *U* test.

### Prognostic significance of TBIL, DBIL, IBIL, and albumin

Five-year overall survival rates and univariate log-rank test results for clinical variables in the training and validation cohorts are shown in Table 6. Five-year OS was shorter in patients with low TBIL, IBIL, and albumin levels (*P*<0.01). Multivariate Cox regression analysis was then conducted for clinical features identified as significant in the univariate log-rank test. T, N, and M stages as well as TBIL and albumin levels were independently prognostic factors for OS in both the training and validation cohorts (Table 6).

**Table 6:** Univariate and multivariate analyses of prognostic significance of serum bilirubin and albumin levels

	Training cohort					Validation cohort				
Variable		Univariate analysis		Multivariate analysis			Univariate analysis		Multivariate analysis	
	5-year OS (%)	Log rank χ^2^ test	*P*	HR (95%CI)	*P*	5-year OS (%)	Log rank χ^2^ test	*P*	HR (95%CI)	*P*
Age		3.543	0.060				2.379	0.121		
<65 years	42.4					34.1				
≥65 years	33.7					28.1				
Gender		1.552	0.213				0.361	0.548		
Male	36.7					29.8				
Female	40.9					32.7				
Smoking		1.683	0.195				0.338	0.561		
Never	35.7					31.2				
Yes	45.0					27.8				
Drinking		0.949	0.330				0.580	0.446		
Never	37.1					29.7				
Yes	43.4					34.2				
*Helicobator pylori*		1.521	0.218				0.001	0.972		
Negative	34.2					28.8				
Positive	40.8					31.8				
Differentiation		33.100	<0.001				18.475	<0.001		
Well	64.0			Reference		57.7			Reference	
Moderate	34.7			1.028 (0.710-1.577)	0.692	30.7			1.442 (0.819-2.539)	0.205
Poor	29.1			1.147 (0.600-1.668)	0.823	22.4			1.617 (0.924-2.828)	0.092
pT stage		153.793	<0.001				96.193	<0.001		
T1	87.0			Reference		80.6			Reference	
T2	66.0			1.022 (0.962-3.605)	0.068	42.9			1.896 (1.232-2.833)	0.011
T3	30.7			1.920 (1.041-4.224)	0.012	32.1			1.937 (1.181-3.280)	0.003
T4	13.5			2.446 (1.253-5.233)	<0.001	9.9			2.341 (1.214-3.676)	<0.001
pN stage		190.446	<0.001				58.345	<0.001		
N0	80.2			Reference		63.9			Reference	
N1	36.6			1.387 (1.056-2.900)	0.043	29.7			1.735 (1.013-2.973)	0.045
N2	17.5			2.006 (1.310-4.018)	<0.001	18.0			2.150 (1.182-3.911)	0.012
N3	10.3			3.043 (1.085-6.364)	<0.001	9.8			2.609 (1.451-4.693)	<0.001
Metastasis		160.020	<0.001				144.813	<0.001		
Absent	53.7			Reference		45.5			Reference	
Present	11.6			1.859 (1.254-2.706)	<0.001	7.8			2.168 (1.407-3.154)	<0.001
Chemotherapy		0.109	0.742				0.168	0.682		
No	40.7					30.2				
Yes	37.8					30.9				
Treatments		98.528	<0.001				120.314	<0.001		
Curative	50.6			Reference		42.7			Reference	
Palliative	15.0			1.556 (1.160-2.011)	<0.001	9.4			2.590 (1.423-3.154)	<0.001
TBIL (μmol/L)		7.613	0.006				31.756	<0.001		
≤5.3	23.5			Reference		7.9			Reference	
>5.3	39.5			0.688 (0.491-0.959)	0.022	36.1			0.794 (0.552-0.951)	0.032
DBIL (μmol/L)		2.204	0.138				0.097	0.756		
≤7.3	38.8					30.0				
>7.3	29.4					36.1				
IBIL (μmol/L)		9.818	0.002				5.099	0.024		
≤6.7	30.8			Reference		25.0			Reference	
>6.7	46.1			0.994 (0.792-1.290)	0.860	37.9			0.915 (0.662-1.264)	0.590
Albumin (g/L)		23.351	<0.001				17.036	<0.001		
≤33.2	22.8			Reference		20.2			Reference	
>33.2	41.8			0.685 (0.520-0.890)	0.002	33.4			0.774 (0.554-0.961)	0.034

TBIL, Total bilirubin; DBIL, direct bilirubin; IBIL, indirect bilirubin. HR, hazard ratio; CI, confidence interval.

### Nomogram for predicting gastric cancer outcomes

**Figure 2:**
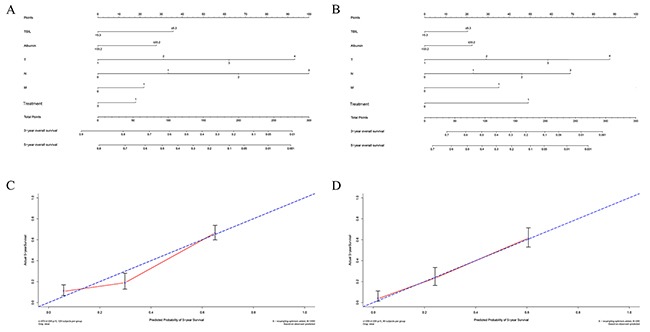
Nomogram for predicting gastric cancer outcomes The sum of the points assigned to each factor by the nomogram is shown at the top of scale. Total point values were used to predict 5-year probability of death in the lowest scale. The c-indexes values for the training cohort **(A)** and the validation cohort **(B)** are 0.762 and 0.744, respectively. Calibration curves for 5-year OS, which are indicative of predictive accuracy, for the training cohort **(C)** and the validation cohort **(D)**. The 45-degree reference line represents a perfect match between observed and predicted values.

To further assess the predictive ability of TBIL and albumin in gastric cancer patients, we used a nomogram based on the results of the univariate analyses to predict 5-year overall survival rates. Tumor grade, T, N, and M stage, treatment approach, and TBIL, IBIL, and albumin level were included in the nomogram for both the training (Figure 2A) and validation (Figure 2B) cohorts. The predictive models for both cohorts showed that treatment with palliative operation and advanced T, N, and M stage were poor prognostic indicators, while high TBIL and albumin levels were favorable factors. These findings were similar to those obtained previously in the multivariate Cox regression analyses (Table 6). Calibration curves for the nomogram in both cohorts revealed that the predicted 5-year OS values were similar to the actual 5-year OS (Figure 2C and 2D).

We then compared the predictive accuracy of the nomogram to that of the TNM staging system (T, N, and M stage only) in the training and the validation cohorts using Harrell's c-index. The Harrell's c-index values for the nomogram in the training and the validation cohorts were 0.774 and 0.760, respectively, compared to 0.727 and 0.702, respectively, for the TNM staging system. Collectively, the predictive accuracy of the nomogram based on TBIL and albumin levels was better than that of the TNM staging system in both patient cohorts (*P*<0.01).

## DISCUSSION

In this study, we confirmed the prognostic significance of serum bilirubin and albumin levels and, to our knowledge, demonstrated for the first time that elevated pre-treatment levels of these factors were positive prognostic factors for survival in gastric cancer. Elevated TBIL and albumin levels were associated with tumor progression and acted as protective prognostic factors in both training and validation cohort gastric cancer patients. Our nomogram also confirmed the prognostic significance of TBIL and albumin in gastric cancer patients.

Bile acid, which is the end product of cholesterol breakdown, exists in three forms in peripheral blood: TBIL, DBIL, and IBIL. Due to its anti-inflammation, antioxidant, and antiproliferation effects, bilirubin acts as a protective factor against carcinogenesis [12]. Decreased serum bilirubin is associated with an increased risk of cancer and with poorer outcomes [7]. Li *et* *al*. demonstrated that non-small cell lung cell cancer patients with higher pretreatment bilirubin levels had longer OS, DFS, and DMFS than those with lower levels [8]. Zhang and colleagues also found that high DBIL was strongly associated with worse outcomes after surgery in colorectal cancer patients with stage II and stage III disease in a retrospective study [9]; subsequent studies confirmed these results [13, 14]. However, the association between serum bilirubin levels and clinical outcomes in gastric cancer had not been examined. Here, we found that decreased serum TBIL was associated with advanced gastric cancer and poor prognosis, as is the case in other types of cancer [8, 9, 15]. Although training and validation cohort patients in our study differed with regard to drinking behavior, correlation analysis revealed no association between drinking status and serum bilirubin and albumin levels. Furthermore, serum TBIL and albumin were independent prognostic indicators regardless of drinking status both in the training and the validation cohorts. Taken together, these results suggest that serum bilirubin may have predictive value in gastric cancer.

Serum albumin, which is commonly used to evaluate an individual's nutritional status, also has antioxidant effects and acts as a transporter of key nutrients. Serum albumin levels fall sharply in patients with advanced cancer due to malnutrition and systemic inflammatory responses to malignancy [16]. Malnutrition can also cause a series of detrimental clinical effects and thus reduce treatment response. Previous studies have assessed the association between serum albumin and survival in gastric cancer patients [17, 18]. Liu *et* *al*. reported that serum albumin was an independent prognostic factor for worse outcomes in 1320 gastric cancer patients after curative resection [19]. In a separate study of 320 gastric cancer patients, Liu *et* *al*. found that preoperative albumin, BMI, and triglyceride levels were more accurate for predicting survival than the TNM system [20]. Our findings strongly suggest that low serum albumin levels are a prognostic indicator of poor outcome in gastric cancer patients, which is consistent with previous findings for other malignancies [21, 22].

Although individual serum markers are useful prognostic factors in studies of cancer patients, single markers may not be adequate for predicting survival in a clinical setting. Combining several markers in a single index may improve their predictive power [23–25], and nomograms are a useful method for combining clinical characteristics to improve survival predictions for individual patients. In the current study, we constructed a nomogram to predict survival in training and validation cohort gastric cancer patients based on several clinical characteristics. The nomogram accurately predicted 5-year overall survival in the training and validation cohorts; Harrell's c-indexes confirmed the accuracy of these predictions. Furthermore, serum TBIL and albumin levels were incorporated into the nomogram using a stepwise algorithm, and the predictive ability of the nomogram was confirmed using calibration curves. Additionally, a comparison of the predictive abilities of the constructed nomogram and the TNM staging system revealed that the nomogram was superior to TNM in both cohorts.

A major strength of this study is that it involved a relatively large number of gastric cancer patients undergoing treatment at a single center and that it included both prospective and retrospective cohorts. However, external validation studies should be performed to determine whether our results are applicable in other patient populations [26]. Another strength of this study is the use of X-tile software, which is a robust graphical tool [27], to determine the optimal cutoff values for serum bilirubin and albumin levels. However, some limitations should be considered when interpreting these results. For example, only pretreatment serum bilirubin and albumin levels were included in the present analyses, and it is possible that dynamic changes in serum bilirubin and albumin levels during the course of treatment might also influence outcomes in gastric cancer patients.

In summary, in this study of 778 gastric cancer patients, we found that a nomogram based on serum TBIL and albumin levels was more accurate than the TNM staging system in predicting survival. These findings suggest that serum TBIL and albumin levels in combination might improve outcome predictions for gastric cancer patients.

## MATERIALS AND METHODS

### Study population

The patients involved in this study were divided among two cohorts. The prospective training cohort consisted of 479 patients who underwent surgical resection or chemotherapy at the Nanjing First Hospital, Nanjing Medical University between January 2010 and November 2012. The retrospective validation cohort consisted of 299 patients who underwent surgical resection or chemotherapy at the same institution between January 2006 and December 2009. All individuals were diagnosed with biopsy-proven gastric adenocarcinoma. Patients who died within 30 days of surgery or who received preoperative antitumor treatment were excluded. Patients with tumors that invaded the biliary tract were also excluded due to the resulting elevation of serum bilirubin levels. All individuals received either open or laparoscopic surgery for either curative or palliative purposes according to the Japanese Classification of Gastric Cancer Guidelines [28]. Detailed clinical characteristics were collected before cancer treatment. Individuals were restaged according to the 7^th^ edition of the American Joint Committee on Cancer staging system.

Regular follow-ups, which occurred every 3 months for the first 2 years and then every 6 months for 3 more years, began after the date of treatment and continued until September 30, 2016 or until patient death. Routine examination, gastroscopy, and imaging were performed at every visit. The follow-up period ranged from 91 to 3535 days, with a median of 1026 days. The study was approved by the Institutional Review Board of Nanjing First Hospital. Informed consent for use of their data was obtained from training cohort patients because of the prospective nature of the study.

### Detection of serum bilirubin and albumin

Blood samples were collected from patients and serum was separated by centrifugation. Serum albumin levels were measured using a Roche Modular D/P automated analyzer (Roche, USA). Serum bilirubin levels were determined using the vanadium oxidation method.

### Statistical analysis

Data assessment was performed using SPSS 20.0 version (SPSS Inc., Chicago, IL, USA) and R 3.3.1 software (Institute for Statistics and Mathematics, Vienna, Austria). First, optimal cutoff values for total bilirubin (TBIL), direct bilirubin (DBIL), indirect bilirubin (IBIL), and albumin levels were determined using X-tile 3.6.1 software (Yale University, New Haven, CT, USA) [27]. Differences between high- and low-level group patients were evaluated using the χ^2^ test or Mann-Whitney *U* test. Survival analysis was performed using the Kaplan-Meier curve and log-rank test. Significant prognostic predictors from the survival analysis were included in a multivariate analysis using the Cox proportional hazards model. The nomogram for significant factors associated with 5-year overall survival (OS) was constructed using R software via a stepwise algorithm, and Harrell's concordance index (c-index) was used to compare the performance of the nomogram to that of the TNM staging system. A calibration curve comparing observed outcomes to predicted outcomes was generated to further evaluate the nomogram's accuracy in predicting prognosis. *P* values of less than 0.05 were regarded as statistically significant.
